# Maternal and perinatal outcomes and pharmacological management of Covid-19 infection in pregnancy: a systematic review protocol

**DOI:** 10.1186/s13643-020-01418-2

**Published:** 2020-07-18

**Authors:** Binny Thomas, Abdulrouf Pallivalapila, Wessam El Kassem, Asma Tarannum, Fatema Al Hail, Mohammed Rijims, Hussain Parappil, Arabo Ibrahim Bayo, Shamsa Ahmad, Zachariah Nazar, Derek Stewart, Moza Al Hail

**Affiliations:** 1 Pharmacy Executive Office, Hamad Medical Corporation, Doha, Qatar; 2 Women’s Wellness and Research Center, Hamad Medical Corporation, Doha, Qatar; 3 Neonatal Intensive Care Unit, Hamad Medical Corporation, Doha, Qatar; 4 College of Pharmacy, QU Health, Qatar University, Doha, Qatar

**Keywords:** COVID-19, Maternal, Perinatal, Pharmacological management, Pregnant

## Abstract

**Background:**

Over 4.2 million confirmed cases and more than 285,000 deaths, COVID-19 pandemic continues to harm significant number of people worldwide. Several studies have reported the impact of COVID-19 in general population; however, there is scarcity of information related to pharmacological management and maternal and perinatal outcomes during the pandemic. Altered physiological, anatomical, and immunological response during pregnancy makes it more susceptible to infections. Furthermore, during pregnancy, a woman undergoes multiple interactions with the health care system that increases her chance of getting infected; therefore, managing pregnant population presents a unique challenge.

**Research questions:**

This systematic review seeks to answer the following questions in relation to COVID-19:

- What are the different clinical characteristics presented in maternal and perinatal population?
- What are the different maternal and perinatal outcome measures reported?
- What are the distinct therapeutic interventions reported to treat COVID-19?
- Is it safe to use “medications” used in the treatment of COVID-19 during antenatal, perinatal, postnatal, and breastfeeding?

**Method:**

The search will follow a comprehensive, sequential three step search strategy. Several databases relevant to COVID-19 and its impact on pregnancy including Medline, CINAHL, and LitCovid will be searched from the inception of the disease until the completion of data collection. The quality of this search strategy will be assessed using Peer Review of Electronic Search Strategies Evidence-Based Checklist (PRESS EBC). An eligibility form will be developed for a transparent screening and inclusion/exclusion of studies. All studies will be sent to RefWorks, and abstraction will be independently performed by two researchers. Risk of bias will be assessed using Cochrane Risk of Bias tool for randomized controlled trials, Newcastle–Ottawa Quality Assessment Scale for non-randomized studies, and for case reports, Murad et al. tool will be used. Decision to conduct meta-analysis will be based on several factors including homogeneity and outcome measures reported; otherwise, a narrative synthesis will be deemed appropriate.

**Discussion:**

This systematic review will summarize the existing data on effect of COVID-19 on maternal and perinatal population. Furthermore, to the best of our knowledge, this is the first systematic review addressing therapeutic management and safety of medicines to treat COVID-19 during pregnancy and breastfeeding.

**Systematic review registration:**

This systematic review has been registered and published with Prospero (CRD42020172773).

## Background

The novel coronavirus now known as SARS-CoV-2 (severe acute respiratory syndrome coronavirus 2) has spread globally [1]. On 12 March 2020, the World Health Organization (WHO) declared it as a “pandemic” outbreak of utmost international concern [2, 3]. The first coronavirus patient was identified in Wuhan City, China, in December 2019 [4, 5]. Previous infectious outbreaks, such as, H1N1 influenza virus, Zika virus, severe acute respiratory syndrome corona virus (SARS-CoV), and Middle East respiratory syndrome corona virus (MERSCoV), have had significant impact on maternal as well as perinatal outcomes [6, 7].

### Epidemiology, transmission, and symptoms

Ever since the first case reported in China, the infection has been spreading at an accelerating pace, across 213 countries, infecting over 3.7 million people, 261,380 cases of death (as of 5 May, 2020), at an estimated case fatality ranging 0.5–6% [3]. Adults and older people are more susceptible to the infection in comparison to younger age group, while the infection is predominantly reported among men, the rate of infection among children is scarce [8, 9]. Furthermore, co-morbidities such as diabetes, hypertension, cardiovascular, or respiratory disorders are significantly associated with poor outcomes and substantially increased mortality rate [10, 11].

While vast majority of cases are categorized as “mild” or “uncomplicated,” almost 14% of patients require hospitalization and/or oxygen support, and approximately 5% required intensive care admission to survive [12]. Common clinical manifestations reported among hospitalized patients were fever (83–100%), cough (59–82%), myalgia (11–35%), headache (7–8%), and diarrhea (2–10%). Almost all patients had abnormal radiographic chest imaging [5, 13, 14].

The National Institutes of Health (NIH) in the USA has since developed a tool to categorize the severity of the disease, based on the presenting symptoms (Table 1).

**Table 1:** NIH COVID-19 severity assessment scale

Severity	Symptoms
Asymptomatic	Positive test for SARS-CoV-2 but no symptoms
Mild illness	Any signs and symptoms (e.g., fever, cough, sore throat, malaise, headache, muscle pain) without shortness of breath, dyspnea, or abnormal chest imaging.
Moderate illness	Evidence of lower respiratory disease by clinical assessment or imaging and a saturation of oxygen (SaO2) > 93% on room air at sea level.
Severe illness	Respiratory frequency > 30 breaths per minute, SaO2 ≤ 93% on room air at sea level, ratio of arterial partial pressure of oxygen to fraction of inspired oxygen (PaO2/FiO2) < 300, or lung infiltrates > 50%.
Critical illness	Respiratory failure, septic shock, and/or multiple organ dysfunction.

The transmission routes of COVID-19 include direct transmission, droplet inhalation transmission, and contact transmission [15].

### COVID-19 and its impact on pregnancy

The “obstetric” population is considered vulnerable as different stages of pregnancy involve multiple interactions with the health care system; therefore, managing pregnant population presents a unique challenge during this pandemic. Additionally, the physiological changes and partial immune suppression during pregnancy makes pregnant women and newborn babies susceptible to several infections. Post-partum hemorrhage, maternal sepsis, preeclampsia, and premature rupture of membrane are the most common COVID-19-induced adverse events reported among pregnant women [16].

Anecdotal evidence suggests that pregnant women do not appear to be different than the general population in terms of disease transmission, and to date, there is no evidence of vertical transmission from mother to fetus.

There are currently limited data on first trimester or early pregnancy COVID-19 infections. One case from Iran reported poor maternal outcomes at 30-week gestation causing maternal and fetal death [17]. Data from the Intensive Care National Audit and Research Centre in the UK reported a marginal difference in the rate of current/recent pregnancy among all individuals admitted to critical care (2.3%) compared to the reported rate for non-COVID viral pneumonia during 2017–19 (3.3%). In one study, 47% (15/32) of pregnant women with COVID-19 infections delivered preterm; another study reported 7/15 women delivered preterm by Cesarean section [17]. Cesarean section is the most commonly reported mode of delivery for patients confirmed with COVID-19 [17]. Several studies have shown no detrimental effect on the fetal growth where COVID-19 positive pregnant women deliver within 13 days after the onset of symptoms [18–21].

Another study followed 13 pregnant women diagnosed with COVID19, mostly in their third trimester (11 patients < 28 weeks gestation). Three were discharged and continued to their pregnancy; however, eight underwent cesarean section due to reasons including fetal distress (3), premature rupture of the membrane or PROM (1), stillbirth (1), multiple organ dysfunction (1), and 6 cases delivered preterm [18]. While in the state of pregnancy, reduced mobility and hospital admissions are considered as risk factors for hypercoagulable disorders; COVID-19 infection during pregnancy is more likely to cause maternal venous-thromboembolism [16].

To date, there is no valid evidence suggesting COVID-19 infection in the amniotic fluid, umbilical cord blood, or breastmilk samples.

### COVID-19 and its impact on perinatal and breastfeeding

While there is scarcity of evidence demonstrating effect of COVID-19 on the fetus, factors such as mother-to-child vertical transmission and its long-term and short-term effect on the offspring remain unclear. Wang et al. reported a case with positive qRT-PCR in both the mother and the neonate. The mother was admitted at 40 weeks gestation, and computerized tomography (CT) images were remarkable, suggesting for COVID-19; following which, she underwent emergency cesarean section delivering the baby with normal Apgar scores. Hours after birth, the neonate had lymphocytopenia, deranged liver function, elevated creatine kinase, and a pharyngeal swab collected 36 h after birth turned positive [20].

### Pharmacological management of pregnant women with COVID-19

Ever since the outbreak, the healthcare team and researchers across the globe have proposed several pharmacological interventions that could prove effective in treating COVID-19 [22]. Various treatment options include, but are not limited to, antivirals and combinations, angiotensin receptor blockers, antimalarial, antihistamines, steroids, and antipyretics [23]. While COVID-19 treatment strategies vary across different countries, the WHO and the Centers for Disease Control and Prevention (CDC) guidelines are very general, and both advise to manage pregnant and pediatric patients with caution.

### Rationale for this review

Infecting over 4 million people, the global pandemic is anticipated to affect considerable number of pregnant women worldwide. The alarmingly increasing mortality rates warrant an early identification and protection of this vulnerable population. Despite favorable maternal and perinatal outcomes reported in most cases, the scientific evidence in managing this vulnerable population remains unclear [24]. There is a scarcity of evidence demonstrating pharmacological management and maternal and perinatal outcomes during COVID-19.

The existing studies have predominantly originated from one geographical location, or as single case or case series. Furthermore, fewer sample size, varied findings, and continuously evolving treatment protocols and ambiguity in reliability of these results make the findings difficult to interpret. Hence, collating scientific evidence concerning the pandemic is imperative. Therefore, a systematic review evaluating the maternal and perinatal outcomes and therapeutic management of pregnant women diagnosed with COVID-19 is highly recommended.

## Research questions

This systematic review seeks to answer the following questions in relation to COVID-19:

- What are the different clinical characteristics presented in maternal and perinatal population?
- What are the different maternal and perinatal outcome measures reported?
- What are the distinct therapeutic interventions reported to treat COVID-19?
- Is it safe to use “medications” used in the treatment of COVID-19 during antenatal, perinatal, postnatal, and breastfeeding?

## Methods/design

### Study registration

This systematic review was registered on the International Prospective Register of Systematic Reviews (PROSPERO) (CRD42020181163) on the 23rd of April 2020 [25] (Additional file 1). This protocol is prepared in accordance with the Preferred Reporting Item for Systematic Review and Meta-analysis (PRISMA-P) statement [26] (Additional file 2); the PROSPERO record will be updated simultaneously in case of any important amendments.

### Eligibility criteria

#### Inclusion

##### Type of studies

Study designs, particularly case reports, case series, observational studies, randomized and quasi-randomized controlled trials (RCTs/CCTs), controlled before and after studies (CBAs), and interrupted time series (ITSs), will be included. Previous systematic reviews will be analyzed for cross-referencing. Only studies published in English will be included in this review.

##### Type of participants

The current review will include all studies reporting clinical characteristics, outcomes, treatment options, and any reported adverse events, in pregnant women diagnosed with COVID-19 and/or the fetuses and infants of mothers who are COVID-19 positive. Infants and children exposed in utero during delivery or breastfeeding will be included. There will be no restriction with respect to age, ethnicity, setting, or location.

##### Type of exposure

Pregnant women infected and diagnosed (laboratory-confirmed infection) with COVID-19 infection (exposure) will be considered as an intervention.

##### Type of comparators

We do not anticipate any RCTs for pregnant women diagnosed with COVID-19; however, if any, uninfected pregnant women will be the comparator/control.

### Outcome measures

#### Outcome 1 (clinical characteristics)

Clinical characteristics as illustrated by the NIH definition will be classified as mild, moderate, and severe. See Table 1.

#### Outcome 2 (maternal and perinatal outcomes)

The maternal mortality and morbidity; other complications such as post-partum hemorrhage, maternal sepsis, and preeclampsia; mode of delivery; PPROM; ventilator support; or ICU. Perinatal outcomes, APGAR scores, neonatal birth outcomes, birth weight, infections, IUGR, and mortality.

#### Outcome 3 (safety of therapeutic interventions)

The primary outcome in terms of safety of the medications used in the treatment of COVID-19 in pregnancy, post-pregnancy, and breastfeeding is presence of malformation, still birth, or child death. Secondary outcome includes drug interactions, drug disease interactions, any adverse drug reactions, other minor adverse drug events, or medication errors reported due to the pharmacological agents used in the treatment of COVID-19 infections among pregnant women.

### Exclusion

Studies that do not report/present a laboratory-confirmed infection will be excluded. Studies published in any other language other than English will be excluded. Studies reporting COVID-19 infections in non-pregnant or women with gynecological complications will excluded from the review. Data obtained from narrative reviews, abstracts, personal opinions, commentaries, and conference presentations will be excluded. Also, studies published before 12 December 2019 will be excluded.

### Databases and search strategy

#### Literature search

The search will be developed by DS and BT and will follow a comprehensive, sequential three step search strategy. Both DS and BT have substantial experience in systematic reviews and developing search strategies. The quality of this search strategy will be assessed by at least two independent researchers using the Peer Review of Electronic Search Strategies Evidence-Based Checklist (PRESS EBC) [27].

The first step will be an initial limited search that will include two most relevant online databases (MEDLINE/PubMed and CINAHL); we will identify the key words in the title and abstract of retrieved papers and the index terms used to describe the articles.

In the second search, we will use all identified keywords and index terms to search across different databases. All search terms used for the search will be added in the appendix. Various Boolean operators (AND/OR/NOT), truncation, wildcards, etc. either individually or in combination will be used to ensure the comprehensiveness of the search process.

The electronic databases that will be searched are as follows:

1. MEDLINE®
2. PubMed®
3. The Cumulative Index of Nursing and Allied Health Literature (CINAHL®)
4. LitCovid
5. ScienceDirect
6. The Cochrane Database of Systematic Reviews (CDSR)
7. The Centre for Reviews and Dissemination (CRD)
8. Joanna Briggs Institute Library (JBI)

Thirdly, the reference list of identified, relevant articles will be searched for additional studies. In case of missing information/incomplete data, the authors of primary studies will be contacted.

All studies published in English from the inception of this disease (December 2019) until the completion of this review will be included. References will be managed using RefWorks.

### Study selection

A study eligibility form will be developed for screening the titles, abstracts, and potentially relevant full-text articles. The form will be developed by BT and DS and shared across the team; the form will be tested and revised if necessary. Inter-rater reliability will be calculated from the pilot-test, and screening will only commence after high agreement (e.g., kappa statistic ≥ 60%) is observed. All the articles will be individually screened by 2 authors, BT/DS; any disagreements will be clarified by contacting a third reviewer (PAR). The studies will be classified into three categories: (1) included, (2) excluded, and (3) pending. Pending articles will be considered as those articles that initially appear to incompletely fit the exclusion and/or the inclusion criteria. A decision to include or exclude the pending articles will be made through discussion and mutual consensus. All screening procedures will be presented using a PRISMA flow diagram.

### Data extraction

All studies identified will be sent to RefWorks and the duplicated will be removed. Data abstraction will be performed by two independent reviewers (BT and PAR) using a standardized template to independently apply the inclusion and exclusion criteria, any disagreements will be resolved by discussion between the two, or else a third reviewer (FAH) will be contacted if no consensus is reached. The extraction will include four main categories:

(i) General characteristics of the reviewed studies such as country, month/year of publication, study objective(s), study design, period of study, and type of hospital, number of beds, and number of patients
(ii) Maternal outcomes: number of patients, age, parity, pregnancy outcome, ethnicity, presence of comorbidities, and any other pregnancy-related complications, maternal mortality, and other outcome-related data

Perinatal outcomes: gestational age, neonatal compromise (APGAR scores), neonatal birth outcomes, birth weight, infections, mal-presentation, intrauterine growth restriction and mortality, the study will also include data on vertical transmission if available.

(iii) Pharmacological interventions: drug name, pharmacological class, dose, route, frequency, concurrent medications used, etc.
(iv) Safety of medications used: if any reported drug interactions, adverse drug reactions, adverse drug events, or any other complications due to pharmacological agents used

### Assessment of risk of bias

In order to achieve a consistency in assessing the risk of bias, the review team will pre-assess a sample of selected studies, and the result of the assessment will be shared among the assessors as well as the review team and discussed. Pairs of reviewers will independently assess quality of each of the studies (BT and PAR). The Cochrane Risk of Bias tool [28] will be used for randomized controlled trials (if any), and for non-randomized studies including retrospective cohort, cohort before and after studies, case control studies, we plan to use the Newcastle–Ottawa Quality Assessment Scale (NOS) [29]. NOS is a three-dimensional appraisal tool to assess the methodological quality that includes (i) selected population, (ii) comparability of groups, and (iii) outcome of interest. Considering scarcity of information around this topic, no studies will be excluded based on the assessment of risk of bias. The study team will also consider Murad et al. tool for assessing quality of case reports and case series [30].

### Data synthesis and analysis

If the studies are found heterogeneous, a narrative synthesis method will be considered as appropriate, all included studies will be summarized in the form of a detailed commentary in accordance to the data extraction form summarized in the previous section. Tables and graphs will be created to illustrate the key study characteristics such as population characteristics, maternal or perinatal outcomes, sample sizes, settings, medications used, results, and any other important aspect related to each research question of interest. Descriptive analysis will be performed for all categorical variables using frequency, mean, and standard deviation wherever possible. If the studies are found similar enough to be pooled, a random effect meta-analysis will be applied. We will consider clinical, methodological, and statistical aspects to determine the heterogeneity among the studies. Variance in the clinical interventions and outcomes studied among the studies will be described as clinically heterogeneous. Methodological heterogeneity will be taken into account if the studies vary in study design or risk of bias. Any variations in the interventions being evaluated will be considered as statistically heterogeneous. Statistical heterogeneity between studies will be assessed using *τ*^2^ and *I*^2^ statistics, and *P* < 0.10 and *I*^2^ > than 50% will be considered as a high level of statistical heterogeneity between the studies.

### Quality of evidence

Two authors will independently assess the quality of evidence for each outcome. The quality of evidence will be determined by Grading of Recommendations Assessment, Development, and Evaluation (GRADE) system [31]. The studies will be rated based on their evidence ranging high, moderate, low, or very low level. Individual outcomes will be assessed for the following aspects, limitations, inconsistency, indirectness, imprecision, and publication bias.

## Discussion

As pandemic continues to affect a large number of pregnant women worldwide, the findings of this systematic review will contribute to improve to the current state of knowledge about effect of COVID-19 on maternal and perinatal population. Furthermore, to the best of our knowledge, this is the first systematic review addressing therapeutic management and safety of medicines to treat COVID-19 during pregnancy and breastfeeding.

Pooling of data for meta-analysis seems difficult as we anticipate a considerable degree of heterogeneity between the studies in terms of study design, exposure measurement, characteristics of participants, and outcomes.

To ensure a wider dissemination, we will present the interim findings at local and international conferences and publish the findings in high impact open access journals. Furthermore, we will brief the findings to key stakeholders, policymakers, antimicrobial stewardship committee, hospital infection control committee, etc.

### Strengths and limitations of this study

- To the best of our knowledge, this will be the first review to use a validated systematic and transparent methodological process to appraise and summarize the existing literature on COVID-19-associated pregnancy and perinatal outcomes.
- To the best of our knowledge, this systematic review is the first to address the pharmacological management in COVID-19 and its impact on maternal and perinatal outcomes.
- The proposed systematic review is in accordance to the Preferred Reporting Items for Systematic Reviews and Meta Analyses guidelines (PRISMA), ensuring consistency and uniformity in reporting the full systematic review.
- Two independent reviewers will screen the eligible studies and perform extraction and quality assessment to minimize potential reviewer bias.
- One key limitation of this review is it will only include studies published in English, while majority of cases were reported from China, and there are chances that we may miss information published in Chinese or other regional languages. This limitation may cause bias.
- There is a possibility of reporting bias, as we plan to exclude no studies based on the quality, and there is a possibility that findings originate from low and inconsistent studies.

This systematic review is anticipated to summarize the available evidence that examined the impact of COVID 19 infections in maternal and perinatal outcomes. Finally, the results will be most likely to be used to provide recommendations to the current topic of concern and provide endorsements to future research.

## Supplementary information

**Additional file 1.** PROSPERO

**Additional file 2.** PRISMA 2009 Checklist

## Abbreviations

SARS-CoV: Severe acute respiratory syndrome corona virus
COVID-19: Coronavirus disease 2019
PRESS EBC: Peer Review of Electronic Search Strategies Evidence-Based Checklist
NOS: Newcastle–Ottawa Quality Assessment Scale
RT-PCR: Real-time polymerase chain reaction
CDC: Centers for Disease Control and Prevention
PROSPERO: The International Prospective Register of Systematic Reviews
PRISMA-P: The Preferred Reporting Item for Systematic Review and Meta-analysis
