# Supplementary material for: Maternal and perinatal outcomes and pharmacological management of Covid-19 infection in pregnancy: a systematic review protocol
Source: Syst Rev. 2020 Jul 18;9:161. doi: 10.1186/s13643-020-01418-2 (PMC7368633; doi:10.1186/s13643-020-01418-2)
Supplement: Supplementary file 1 — Additional file 1. PROSPERO [file 13643_2020_1418_MOESM1_ESM.pdf]

Clinical characteristics, maternal and perinatal outcomes and pharmacological management during Covid-19: a systematic review protocol

Binny Thomas, Moza Al Hail, Pallivalappila Abdulrouf, Wessam Elkassem, Asma Tarannum, Fatema Al Hail, Mohammed Rijims, Hussain Parappil, Arabo Ibrahim Bayo, Shamsa Ahmad, Zachariah Jamal Nazar, Derek Stewart

Citation

Binny Thomas, Moza Al Hail, Pallivalappila Abdulrouf, Wessam Elkassem, Asma Tarannum, Fatema Al Hail, Mohammed Rijims, Hussain Parappil, Arabo Ibrahim Bayo, Shamsa Ahmad, Zachariah Jamal Nazar, Derek Stewart. Clinical characteristics, maternal and perinatal outcomes and pharmacological management during Covid-19: a systematic review protocol. PROSPERO 2020 CRD42020181163 Available from:
https://www.crd.york.ac.uk/prospero/display_record.php?ID=CRD42020181163

Review question

This systematic review seeks to answer the following questions in relation to Covid-19 and pregnancy,

• What are the different clinical characteristics presented in maternal and perinatal population diagnosed with Covid-19?

• What are the different maternal and perinatal outcome measures reported?

• What are the distinct therapeutic interventions reported to treat Covid-19?

• Is it safe to use anti-retroviral drugs during antenatal, perinatal, postnatal and breastfeeding?

Searches

The systematic review protocol was developed in accordance with the Preferred Reporting Items for Systematic Review and Meta-Analysis Protocols (PRISMA-P) guidelines.

Literature search

The search will follow a comprehensive, sequential three step search strategy,

The first step will be an initial limited search that will include two most relevant online databases (MEDLINE/PubMed and CINAHL), we will identify the key words in the title and abstract of retrieved papers, and the index terms used to describe the articles.

In the second search, we will use all identified keywords and index terms to search across different databases. All search terms used for the search will be added in the appendix. Various boolean operators (AND/OR/NOT), truncation, wildcards etc. either individually or in combination will be used to ensure the comprehensiveness of the search process.

The electronic database that will be searched are,

1. MEDLINE®

2. PubMed®

3. The Cumulative Index of Nursing and Allied Health Literature (CINAHL®)

4. International Pharmaceutical Abstracts (IPA®)

5. PsycINFO®

6. ScienceDirect

7. The Cochrane Database of Systematic Reviews (CDSR)

8. The Centre for Reviews and Dissemination (CRD)

9. The Database of Abstracts of Reviews of Effectiveness (DARE)

10. Joanna Briggs Institute Library (JBI)

Thirdly, the reference list of identified, relevant articles will be searched for additional studies. In case of missing information/incomplete data, the authors of primary studies will be contacted, if required.

Full text articles reporting information about maternal or perinatal outcomes during Covid-19, therapeutic interventions used in treatment and evidence demonstrating its safety during antenatal, perinatal, postnatal or breastfeeding while using covid-19 therapeutic options.

All studies published in English from the inception of this disease (December 2019) until the completion of this review will be included. A detailed inclusion exclusion and PICO is given below.

The quality of this search strategy will be assessed by at least two independent researchers using the Peer Review of Electronic Search Strategies Evidence-Based Checklist (PRESS EBC).

Types of study to be included

Study designs, particularly, case reports, case series, observational studies, randomized and quasi-randomized controlled trials (RCTs/CCTs), controlled before and after studies (CBAs) and interrupted time series (ITSs), will be included. Previous systematic reviews will be analyzed for cross-referencing.

Condition or domain being studied

The novel corona virus infection that first emerged in Wuhan, China, in December 2019 has now been characterized as a ‘pandemic’ and public health emergency of international concern by the World Health Organization. Previous emerging infections such as, H1N1 influenza virus, Zika virus, severe acute respiratory syndrome corona virus (SARS-CoV) and Middle East respiratory syndrome corona virus (MERSCoV) have shown to have significant impact on maternal as well as perinatal outcomes. Thus, the current systematic review will aim to synthesize available evidence behind maternal outcomes (such as mode of delivery, maternal infections or associated complications, morbidity and mortality) and perinatal outcomes (such as Apgar scores, neonatal birth outcomes, birth weight, infections, morbidity and mortality etc). The review will also report different therapeutic intervention used to treat corona virus in pregnancy and synthesize available evidence behind the use of antiviral medications during pregnancy, postnatal and breastfeeding.

Participants/population

The current review will include all studies reporting data on pregnant women diagnosed with covid-19. While the altered physiological and anatomical status during pregnancy make the mother more vulnerable to severe infections, the immaturity and adaptive immune systems among the fetus and the newborn makes them susceptible to contagions.

Population studied – all pregnant patients diagnosed with Covid-19 infection and all their newborns.

Inclusion criteria:

• Pregnant patients diagnosed with covid-19 (using standard criteria)

• Availability of clinical characteristics including maternal and perinatal outcomes

• Any age group or ethnic origin

• Only studies published in English language

Exclusion

• All studies that do not present a laboratory confirmed infection will be excluded.

• Studies published in any other language other than English will be excluded.

• Studies reporting covid-19 infections in non-pregnant or gynecological cases.

• Data obtained from reviews, personal opinions, commentaries, conference presentations will be excluded.

Intervention(s), exposure(s)

Covid-19 infection – the WHO has defined COVID-19 as an infectious disease caused by coronavirus during the recent outbreak in Wuhan, China, in December 2019. This is characterized by fever, tiredness, and dry cough. While some patients may have mild symptoms such as aches and pains, nasal congestion, runny nose, sore throat or diarrhea, other may develop more serious issues such as difficulty breathing.

Antiretroviral medications in Covid-19 treatment: All antiviral medications used in the treatment of Covid-19.

Comparator(s)/control

We do not anticipate any RCTs for pregnant women with covid, however if there are any, outcomes of pregnant women without covid19 infection will be the comparator/control

Main outcome(s)

In terms of outcome measures,

Outcome 1 (clinical characteristics): Clinical characteristics as illustrated by the WHO definition, will be classified as mild, moderate and severe. Mild symptoms will include aches and pains, nasal congestion, runny nose, sore throat or diarrhea, whereas moderate will include fever, tiredness, and dry cough and severe will be categorized as those who encounter difficulty to breathing and or require ventilator support.

Outcome 2 (maternal and perinatal outcomes): The maternal mortality and morbidity, other complications such as post-partum hemorrhage, maternal sepsis, preeclampsia etc., mode of delivery, PPROM, ventilator support or ICU. Perinatal outcomes, APGAR scores, neonatal birth outcomes, birth weight, infections, IUGR and mortality etc.).

Outcome 3 (different therapeutic interventions): to assess the different pharmacological interventions used in the management of covid-19 infections and to demonstrate the pattern of use among studies.

Outcome 4 (safety of therapeutic interventions): to identify the safety of the medications used in the treatment of covd-19 in pregnancy, post pregnancy and breastfeeding, drug interactions, drug disease interactions, any adverse drug reactions, adverse drug events or medication errors reported due to the pharmacological agents used in the treatment of covid19 infections among pregnant women.

* Measures of effect

Percentages and frequency will be used to express disease severity and maternal and fetal outcomes. The results will be expressed as frequency and 95% CI.

Additional outcome(s)

None

* Measures of effect

Not applicable

Data extraction (selection and coding)

All studies identified will be sent to RefWorks and the duplicated will be removed. Data abstraction will be performed by two independent reviewers (BT and PAR) using a standardized template to independently apply the inclusion and exclusion criteria, any disagreements will be resolved by discussion between the two, or else a third reviewer (FAH) will be contacted if no consensus are reached. The The extraction will include four main categories,

(i) General Characteristics of the reviewed studies such as, country, month/year of publication, study objective(s), study design, period of study, and type of hospital, number of beds, and number of patients.

(ii) Maternal outcomes: number of patients, age, parity, pregnancy outcome, ethnicity, presence of comorbidities, and any other pregnancy related complications, maternal mortality, and other outcome related data.

Perinatal outcomes: gestational age, neonatal compromise (APGAR scores), neonatal birth outcomes, birth weight, infections, mal-presentation, intrauterine growth restriction and mortality etc.), the study will also include data on (vertical transmission if available.

(iii) Pharmacological interventions: drug name, pharmacological class, dose, route, frequency, concurrent medications used etc.

(iv) Safety of medications used: if any reported drug interactions, adverse drug reactions, adverse drug events or any other complications due to pharmacological agents used.

Risk of bias (quality) assessment

Pairs of reviewers will independently assess quality of each of the studies (BT & PAR). The Cochrane Risk of Bias tool will be used for randomized controlled trials and for non randomized studies we plan to use the Newcastle–Ottawa Quality Assessment Scale (NOS). NOS is a three-dimensional appraisal tool to assess the methodological quality that includes, (i) selected population, (ii) comparability of groups, and (iii) outcome of interest.

Strategy for data synthesis

As of now a narrative synthesis method is considered most appropriate, all included studies will be summarized in a narrative form. Tables and graphs will be created to illustrate the key study characteristics such as (population characteristics, maternal or perinatal outcomes, sample sizes, settings, medications used, results, and any other important aspect related to each research question of interest). Descriptive analysis will be performed for all categorical variables using frequency, mean and standard deviation wherever possible. If the studies are found similar enough to be pooled, a random effect meta-analysis will be applied Statistical heterogeneity between studies will be assessed using $?^2$ and I^2 statistics and $P < 0.10$ and I^2>than 50%, will be considered as a high level of statistical heterogeneity between the studies.

Analysis of subgroups or subsets

None

Contact details for further information

Binny Thomas
binnyinhmc@gmail.com

Organisational affiliation of the review

Hamad Medical Corporation
hamad.qa

Review team members and their organisational affiliations

Dr Binny Thomas. Hamad Medical Corporation
Professor Moza Al Hail. Hamad Medical Corporation
Dr Pallivalappila Abdulrouf. Hamad Medical Corporation
Dr Wessam Elkassem. Hamad Medical Corporation
Dr Asma Tarannum. Hamad Medical Corporation
Dr Fatema Al Hail. Hamad Medical Corporation

Dr Mohammed Rijims. Hamad Medical Corporation
Dr Hussain Parappil. Hamad Medical Corporation
Arabo Ibrahim Bayo. Hamad Medical Corporation
Dr Shamsa Ahmad. Hamad Medical Corporation
Dr Zachariah Jamal Nazar. Qatar University
Professor Derek Stewart. Qatar University

Type and method of review
Meta-analysis, Narrative synthesis, Systematic review

Anticipated or actual start date
21 April 2020

Anticipated completion date
25 May 2020

Funding sources/sponsors
Medical Research Center, Hamad Medical Corporation

Grant number(s)
Under process

Conflicts of interest
None known

Language
English

Country
Qatar

Stage of review
Review Ongoing

Subject index terms status
Subject indexing assigned by CRD

Subject index terms
COVID-19; Female; Humans; Parturition; Pregnancy; severe acute respiratory syndrome coronavirus 2

Date of registration in PROSPERO
23 April 2020

Date of first submission
21 April 2020

Stage of review at time of this submission

Stage	Started	Completed
Preliminary searches	Yes	No
Piloting of the study selection process	No	No
Formal screening of search results against eligibility criteria	No	No
Data extraction	No	No
Risk of bias (quality) assessment	No	No
Data analysis	No	No

Revision note

We have revised out title and added 2 expert doctors (AB & SA) from obstetrics department and added ZN as he is expert in risk of bias and quality assessment of systematic reviews

The record owner confirms that the information they have supplied for this submission is accurate and complete and they understand that deliberate provision of inaccurate information or omission of data may be construed as scientific misconduct.

The record owner confirms that they will update the status of the review when it is completed and will add publication details in due course.

Versions

23 April 2020
23 April 2020
07 May 2020

PROSPERO
This information has been provided by the named contact for this review. CRD has accepted this information in good faith and registered the review in PROSPERO. The registrant confirms that the information supplied for this submission is accurate and complete. CRD bears no responsibility or liability for the content of this registration record, any associated files or external websites.
